# Validation and Application of an Online Self-Assessment Questionnaire for the Assessment of Perception of Functional Foods: A Cross-Sectional Psychometric Study in Adults

**DOI:** 10.3390/nu17182938

**Published:** 2025-09-12

**Authors:** Leandro Oliveira, Ahmed Othman Alsabih, Hani A. Alfheeaid, Najla A. Albaridi, Sehad N. Alarifi, Nada Alqarawi, Ibrahim Alasqah, António Raposo

**Affiliations:** 1 CBIOS (Research Center for Biosciences and Health Technologies), ECTS (School of Health Sciences and Technologies), Lusófona University, Campo Grande 376, 1749-024 Lisboa, Portugal; 2 Department of Physiology, College of Medicine, King Saud University, Riyadh 11461, Saudi Arabia; aalsabih@ksu.edu.sa; 3 Department of Food Science and Human Nutrition, College of Agriculture and Food, Qassim University, Buraydah 51452, Saudi Arabia; h.alfheeaid@qu.edu.sa; 4 Department of Health Science, College of Health and Rehabilitation, Princess Nourah bint Abdulrahman University, P.O. Box 84428, Riyadh 11671, Saudi Arabia; naalbaridi@pnu.edu.sa; 5 Department of Food and Nutrition Science, Al-Quwayiyah College of Sciences and Humanities, Shaqra University, Shaqraa 11971, Saudi Arabia; snalarifi@su.edu.sa; 6 Department of Psychiatric and Mental Health, and Community Health, College of Nursing, Qassim University, Buraydah 51452, Saudi Arabia; n.alqarawi@qu.edu.sa (N.A.); i.alasqah@qu.edu.sa (I.A.)

**Keywords:** functional foods, food perception, psychometrics, food literacy, consumer attitudes, Portugal

## Abstract

**Background/Objectives:** The increasing interest in functional foods has highlighted the need to better understand consumer perceptions and their influence on dietary behaviours. This study aimed to evaluate the psychometric properties of the Questionnaire for the Assessment of Perception of Functional Foods (QAPAF) and apply it to a Portuguese adult population to explore associations with sociodemographic and lifestyle factors. **Methods:** Participants were recruited through convenience sampling; the achieved sample was predominantly female and highly educated. The 17-item QAPAF was assessed through exploratory factor analysis (EFA), internal consistency (Cronbach’s alpha), and test–retest reliability. Associations between QAPAF scores and participant characteristics were analysed using non-parametric tests. **Results:** EFA supported a four-factor structure, explaining 58.8% of total variance. Internal consistency was acceptable (α = 0.70), and test–retest analysis (*n* = 25) showed no significant score differences, indicating temporal stability. QAPAF scores were significantly higher among participants with higher education and among non-smokers and non-drinkers. No associations were found with sex, BMI, or income. Participants with correct understanding of functional foods were more likely to reject misconceptions and express trust in professional recommendations. **Conclusions:** The QAPAF is a valid and reliable tool for assessing functional food perceptions. Its application provides insights into consumer attitudes and may support the design of targeted food literacy interventions. Generalizability is limited by the convenience sampling and by the predominance of female and highly educated participants; external validation in more diverse samples and cultural contexts is warranted.

## 1. Introduction

Functional foods have received increasing attention over the past two decades due to their potential role in promoting health and preventing disease [1,2,3]. Defined as foods that, beyond their basic nutritional value, exert beneficial physiological effects when consumed as part of a regular diet, they have gained prominence in scientific research, food innovation, and health communication strategies [4,5]. In Europe, regulatory frameworks such as Regulation (EC) No 1924/2006 have established strict criteria for the use of health and nutrition claims, reflecting the need to safeguard consumer protection and ensure informed decision-making [6,7].

Although functional foods are widely available across European markets, consumer acceptance and use remain heterogeneous, influenced not only by factual knowledge, but also by perceptions, attitudes, trust in health information, and broader beliefs about food and health [8,9,10]. Understanding these perceptions is therefore essential for developing public health messages and supporting the effective integration of functional foods into healthy dietary patterns [11,12].

Previous research has shown that consumers generally hold favourable views regarding the benefits of functional foods, especially when these are associated with natural ingredients or traditional diets such as the Mediterranean [13,14]. However, persistent concerns regarding processing, artificiality, cost, and the credibility of health claims often contribute to ambivalence or rejection [15,16]. In addition, confusion surrounding the term “functional”, often conflated with supplements or fortified products, may obscure understanding and limit adoption [17,18]. Sociocultural norms, education, and personal health motivation also play key roles in shaping consumer attitudes and intentions [19,20].

In this context, the concept of food literacy has gained growing importance. It includes not only the ability to access and understand nutritional information, but also the capacity to critically evaluate claims and make informed dietary decisions [21,22,23]. Assessing perceptions of functional foods is thus a crucial step in identifying knowledge gaps, evaluating public trust, and developing targeted communication and education strategies adapted to different population groups [24,25].

Despite the increasing relevance of the topic, few validated tools are available to assess consumer perceptions of functional foods in a multidimensional and culturally sensitive way. In our previous work, we developed the Questionnaire for the Assessment of Perception of Functional Foods (QAPAF) to address this gap [26,27]. Grounded in a theoretical model of food perception, the QAPAF integrates cognitive, affective and behavioural dimensions. In our previous work, we used this instrument in descriptive studies of functional food perception [27]. However, its psychometric properties had not yet been systematically validated, limiting its application in wider surveillance and intervention contexts.

In recent years, the functional food market in Portugal has shown consistent growth, particularly in urban areas and among health-conscious consumers [28,29]. Nonetheless, national data on consumer knowledge, trust, and usage patterns remain scarce. A recent survey by the Directorate-General of Health (Direção-Geral da Saúde) highlighted persistent gaps in food literacy, especially in interpreting nutritional claims, with scepticism towards functional ingredients being particularly marked among older adults and those with lower education [30,31]. Although these products are increasingly present in the retail environment, their integration into daily food practices remains uneven, reinforcing the need for robust tools to monitor perceptions and identify barriers to adoption [32].

Across Europe and other regions, multiple studies have examined how consumers perceive functional foods, identifying facilitators such as health motivation, trust in professional recommendations, and perceived naturalness, and barriers including price, processing, and the credibility of health claims [33,34,35]. Building on this literature, we expected to confirm a four-component structure for the QAPAF with acceptable internal consistency and temporal stability, and to observe higher scores among participants with higher education and health-oriented behaviours. The novelty of this work is threefold: (i) it provides the first comprehensive psychometric validation of the QAPAF in Portuguese adults; (ii) it combines validation with an application that links perception scores to sociodemographic and lifestyle variables; and (iii) it offers an English version and practical scoring guidance to facilitate use in population surveys and intervention evaluation, building on preliminary development work [27].

This study was therefore conducted in two parts. Study 1 aimed to validate the psychometric properties of the QAPAF, including its factor structure, internal consistency, and test–retest reliability in a sample of Portuguese adults. Study 2 applied the instrument to describe consumer perceptions and explore associations with sociodemographic and behavioural variables, with particular attention to conceptual understanding of functional foods and engagement in health-promoting behaviours.

## 2. Materials and Methods

### 2.1. The Psychometric Evaluation of the QAPAF

#### 2.1.1. Participants and Procedures

**Figure 1:**
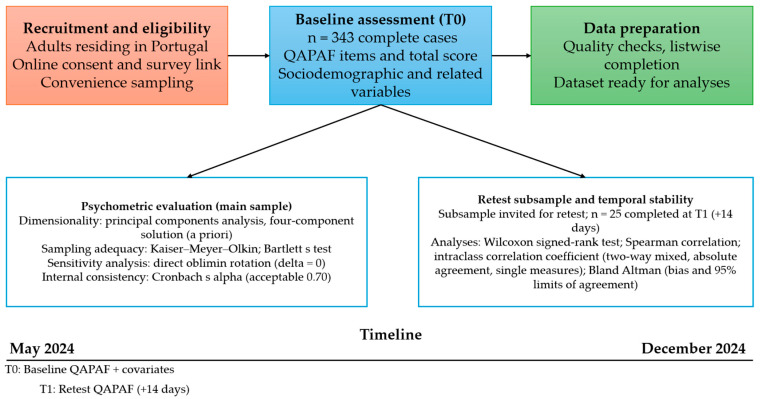
Study design and timeline. Adult participants in Portugal completed the baseline online assessment (T0; *n* = 343 complete cases). Psychometric analyses were conducted in the main sample (PCA, four-component solution; Kaiser–Meyer–Olkin; Bartlett *p* < 0.001), with a sensitivity analysis using direct oblimin rotation. A subsample (*n* = 25) completed a retest after 14 days (T1) for temporal stability analyses (Wilcoxon, Spearman correlation, intraclass correlation coefficient two-way mixed absolute agreement, single measures; Bland–Altman). Abbreviation: QAPAF: Questionnaire for the Assessment of Perception of Functional Foods.

A cross-sectional study was conducted with a convenience sample of 343 adults residing in Portugal. Inclusion criteria included age 18 years or older and proficiency in reading and understanding the Portuguese language. Data collection took place between May and December 2024 using an online questionnaire distributed via Google Forms (Figure 1).

The study followed the principles of the Declaration of Helsinki [36] and was approved by the Ethics Committee of Universidade Lusófona (reference: P04-24, 30 April 2024). Participation was voluntary and anonymous, and informed consent was obtained electronically before beginning the questionnaire. Participants were free to withdraw at any time without consequences.

The sample size was determined based on established methodological recommendations for factor analysis, which suggest using between 10 and 20 participants per item to ensure the statistical robustness of parameter estimates [37]. Considering the 17 items of the QAPAF, the sample of 340 participants was deemed adequate for the psychometric analysis. Study 2 used the same sample as Study 1. The same inclusion criteria and ethical procedures previously described were applied.

#### 2.1.2. Instrument

The initial version of the QAPAF [26] included 17 items, presented in randomised order and rated on a five-point Likert-type scale: 1 = “Strongly disagree”, 2 = “Disagree”, 3 = “Neither agree nor disagree”, 4 = “Agree”, and 5 = “Strongly agree”. An additional response option (“I don’t know”) was available and treated as a neutral response (value = 3). Items 2, 4, 5, 6, 7, 8, 11, 14, 15, and 16 were negatively worded and were reverse-coded during data processing to ensure that higher scores consistently reflected more favourable perceptions of functional foods. The total score, obtained by summing the scores of all 17 items, ranges from 17 to 85. Higher scores indicate more positive attitudes and greater confidence towards functional foods. To minimise interpretation bias, all participants were presented with a standardised definition of functional foods prior to completing the scale. Before answering the QAPAF, all participants were presented with a standardised definition of functional foods to ensure consistent interpretation and minimise bias. The definition was as follows: A functional food is one that, beyond its appropriate nutritional effects, exerts a beneficial effect on one or more body functions, contributing to improved health and well-being and/or reducing the risk of disease. It is consumed as part of a normal diet and not in the form of pills, capsules, or dietary supplements [38]. No brand names or examples were included in the questionnaire to avoid marketing influence. The full list of items and the English version of the QAPAF are provided in the Supplementary Material (Table S1).

#### 2.1.3. Statistical Analysis

Analyses were conducted in IBM SPSS Statistics version 28.0. Descriptive statistics were computed for all items and total scores. Dimensionality was examined with principal components analysis (PCA); the primary unrotated solution is reported to align with the original instrument and to present the partition of total variance. The number of factors was prespecified as four, grounded in the instrument’s conceptual model and the original version. Sampling adequacy was assessed using the Kaiser–Meyer–Olkin (KMO) measure and Bartlett’s test of sphericity. Communalities, eigenvalues, and the scree plot were inspected. As an exploratory check, factor retention followed the Kaiser criterion (eigenvalue > 1) and visual inspection of the scree plot, which were consistent with the prespecified four-factor structure. Loadings ≥ 0.40 were considered salient. Internal consistency was assessed with Cronbach’s alpha (acceptable ≥ 0.70). Corrected item–total correlations and the impact of removing each item on overall reliability were examined [39]. For transparency, a sensitivity analysis with oblique rotation (oblimin) was conducted given plausible correlations among dimensions.

#### 2.1.4. Test–Retest Reliability

Test–retest reliability was assessed in a subsample of 25 participants who completed the questionnaire again after a two-week interval. Although small, this sample size is consistent with previous studies using subsamples of 20–30 individuals for preliminary assessments of temporal stability in similar psychometric contexts [40]. Paired scores were compared with the Wilcoxon signed-rank test; we report the Z statistic, two-tailed *p* value, and the effect size *r* = *Z*/*N*∗, where *N*∗ is the number of non-zero paired differences. Rank-order association was examined with Spearman’s ρ. Absolute agreement was quantified using the intraclass correlation coefficient (ICC) from a two-way mixed-effects model with absolute-agreement definition and single-measures unit, with 95% confidence intervals. Agreement was also visualised with Bland–Altman plots, reporting mean bias and 95% limits of agreement (mean ± 1.96 × SD of the differences) and checking proportional bias by regressing differences on the mean. All tests were two-tailed with statistical significance set at *p* < 0.05 [39].

### 2.2. Application of the QAPAF in the Portuguese Population

#### 2.2.1. Questionnaire Structure

In addition to the QAPAF, the questionnaire included the following variables: Sociodemographic characteristics: sex, age, nationality, marital status, educational attainment, region and type of residence, occupation or field of study, and household income; Anthropometric data: self-reported height and weight, used to calculate Body Mass Index (BMI), classified according to WHO criteria [41]; Health conditions: self-reported chronic illnesses. Before answering the QAPAF, all participants were presented with the same standardised definition of functional foods used in Study 1, to ensure conceptual clarity and consistency across responses. In addition to the QAPAF, the questionnaire included sociodemographic, anthropometric, and health-related variables. As in Study 1, all negatively worded QAPAF items were reverse-coded so that higher scores consistently reflected more favourable perceptions. The total score, ranging from 17 to 85, served as an overall index of functional food perception [26].

#### 2.2.2. Statistical Analysis

Categorical variables were described using frequencies and percentages, while continuous variables were summarised using medians and interquartile ranges (IQR), as normality assumptions were not met (Shapiro–Wilk test). Group comparisons were performed using non-parametric tests: Mann–Whitney U test for continuous or ordinal variables; Chi-square, likelihood ratio, or Fisher’s exact test for categorical variables; Spearman’s correlation for continuous or ordinal variables. The significance level was set at *p* < 0.05. All analyses followed methodological recommendations for non-parametric data in health sciences [39].

## 3. Results

### 3.1. Psychometric Evaluation of the QAPAF

Prior to factor extraction, sampling adequacy was assessed. The Kaiser–Meyer–Olkin (KMO) measure was 0.821, indicating meritorious adequacy for factor analysis. Bartlett’s test of sphericity was statistically significant (χ^2^ (136) = 2002.80, *p* < 0.001), confirming that the correlation matrix was appropriate for factor extraction.

An EFA was performed using principal component analysis without rotation, and the decision to extract four components was theoretically driven, in accordance with the original structure of the QAPAF. All 17 items were retained. The four-component solution explained 58.8% of the total variance (Table 1).

The communalities reflect the proportion of variance in each item explained by the retained components. As shown in Supplementary Table S2, values ranged from 0.30 (Item 17) to 0.75 (Item 3), indicating that all items contributed meaningfully to the factor structure. Most items presented communalities above 0.45, supporting their inclusion in the final model. The scree plot (Supplementary Figure S1) also supported the retention of four components, with a visible inflection point observed after the fourth factor

The total scale demonstrated adequate internal consistency, with a Cronbach’s alpha of 0.70. Most item-total correlations were satisfactory. However, Item 13 and Item 16 showed negative or weak corrected item-total correlations. Despite this, removing these items did not significantly improve reliability, and they were retained to preserve the theoretical consistency and content validity of the questionnaire (Supplementary Table S3).

In a sensitivity analysis using oblique rotation (direct oblimin, delta = 0), sampling adequacy was good (KMO = 0.821), and Bartlett’s test was significant, χ^2^(136) = 2002.796, *p* < 0.001. The four-component solution accounted for 58.8% of the variance and remained conceptually stable. Inter-factor correlations were small in magnitude (|r| ≤ 0.17). Salient pattern loadings (≥0.40) mapped as follows: Component 1 included items 2, 4, 5, 6, 7, 11, and 15; Component 2 included items 12, 16, and 17, with item 9 showing a cross-loading; Component 3 included items 1, 8, and 13; and Component 4 was driven by item 3. One item showed low communality (item 17 = 0.301). Full pattern and structure matrices, the factor correlation matrix, and the scree plot are available in Supplementary Table S4 and Figure S2.

In the retest subsample (*n* = 25), test and retest totals were identical for every participant. The Wilcoxon signed-rank test returned Z = 0 and *p* = 1.000, with 0 negative ranks, 0 positive ranks, and 25 ties; the effect size was r = 0.00. Spearman’s correlation was ρ = 1.00 (*p* not estimable in SPSS under perfect identity). The two-way mixed, absolute-agreement ICC (single measures) was 1.00; confidence intervals and the F test were not estimable for the same reason (Table 2). The Bland–Altman plot showed zero mean bias and zero limits of agreement, reflecting null differences across time points (Supplementary Figure S3).

### 3.2. Application of the QAPAF: Perceptions of Functional Foods

Table 3 presents the distribution of sociodemographic and lifestyle characteristics of participants according to biological sex and their understanding of functional foods. The final sample comprised 343 participants, predominantly female (73.8%), with a median age of 31.0 years (interquartile range [IQR]: 23.0–45.0 years). Most participants reported living in urban areas (82.5%) and resided outside the Lisbon metropolitan area (57.1%). Regarding marital status, 62.4% were married. In terms of education, 61.5% had completed higher education, while 38.5% had completed up to the 12th grade. Among those who provided information on their field of study or professional area (*n* = 213), 26.5% were in health-related areas. The median monthly family income was €2400 (IQR: €1500–€3000). Based on self-reported height and weight, the median Body Mass Index (BMI) was 22.9 kg/m^2^ (IQR: 20.7–25.6), with 67.6% classified as having low or normal weight and 32.4% as overweight. Regarding health status, 39.6% reported having at least one chronic condition or disease. Lifestyle behaviours indicated that 14.9% were smokers, 48.4% reported consuming alcoholic beverages, and 78.6% consumed caffeinated beverages such as coffee or energy drinks.

No statistically significant differences were observed in the understanding of functional foods between males and females (*p* = 0.244). Participants identifying as male were significantly older than females (median age: 37.0 vs. 29.0 years; *p* = 0.041), had a higher prevalence of alcohol consumption (61.4% vs. 38.6%; *p* = 0.025), and reported higher monthly family income (median: €2500 vs. €2000; *p* = 0.030). Males also presented a significantly higher median BMI compared to females (24.6 vs. 22.5 kg/m^2^; *p* = 0.004).

Regarding marital status, a significantly greater proportion of females were married compared to males (66.0% vs. 52.2%; *p* = 0.020). A higher proportion of men resided in Lisbon compared to other regions (*p* = 0.019) and were more frequently working in health-related professional areas (*p* = 0.014). No other statistically significant differences were found in the remaining sociodemographic or lifestyle variables by sex. When stratified by the level of understanding of functional foods, no statistically significant associations were found with any sociodemographic or lifestyle variables. This suggests that knowledge regarding functional foods was relatively evenly distributed across groups regardless of age, sex, education level, BMI, or health-related behaviours.

Table 4 displays the distribution of participants’ responses to a series of statements about functional foods, using a 5-point Likert scale. Most participants agreed that functional foods should be consumed as part of a varied diet, with 39.7% agreeing and 26.2% strongly agreeing. The majority disagreed with the statement that functional foods are useless for healthy individuals (69.7%). Regarding the belief that functional foods can repair damage from an unhealthy diet, 39.7% of participants neither agreed nor disagreed, while 31.8% agreed. Taste was not generally perceived as a barrier, with only 8.1% agreeing or strongly agreeing that functional foods do not taste good.

No statistically significant differences were found in response distributions between male and female participants (*p* > 0.05 for all items). Therefore, only the comparisons according to correctness of functional food understanding are reported.

Three statements revealed statistically significant associations with participants’ level of knowledge: the perception that functional foods are unnecessary (*p* = 0.027), with higher disagreement among those with correct understanding; the belief that functional foods are only for the elderly, sick, or children (*p* = 0.005), where participants with correct knowledge more frequently disagreed; trust in functional foods when recommended by health professionals (*p* = 0.019), with greater agreement among those with correct knowledge.

Overall, responses indicate a favourable perception of functional foods, especially in terms of their safety, effectiveness, and contribution to well-being. However, some scepticism remains regarding advertising claims and perceived cost.

Table 5 presents the association between sociodemographic and lifestyle variables and QAPAF scores. Overall, the median QAPAF score was similar across most subgroups, with no statistically significant differences observed according to sex, marital status, region or type of residence, field of study, body mass index classification, or presence of chronic conditions (*p* > 0.05).

Participants with higher education reported significantly higher QAPAF scores compared to those with education up to the 12th grade (59.0 [55.0–64.0] vs. 58.0 [52.0–61.8]; *p* = 0.010). Similarly, non-smokers scored higher than smokers (59.0 [54.0–64.0] vs. 57.0 [50.5–60.0]; *p* = 0.008), and participants who did not consume alcoholic beverages had higher scores than those who did (59.0 [53.3–64.0] vs. 57.0 [53.0–61.0]; *p* = 0.020).

No significant correlations were found between QAPAF scores and age (r = −0.008; *p* = 0.883), BMI (r = 0.050; *p* = 0.407), or monthly family income (r = −0.058; *p* = 0.408).

## 4. Discussion

This study contributes to the understanding of how functional foods are perceived by adults in Portugal by re-evaluating the psychometric properties of the QAPAF and applying it in a population-based sample. Together, the two studies offer a comprehensive assessment of the instrument’s validity and provide new insights into how knowledge, beliefs, and lifestyle factors influence consumer attitudes towards functional foods [1,42,43].

### 4.1. Psychometric Evaluation of the QAPAF

The findings from Study 1 confirmed the theoretical four-factor model of the QAPAF, encompassing perceived benefits, perceived necessity, trust in efficacy, and safety concerns. These results support the multidimensional nature of consumer perception and are consistent with previous literature that highlights the complexity of how functional foods are understood and evaluated [4,26,44]. The proportion of variance explained (58.8%) and the internal consistency (Cronbach’s alpha = 0.70) indicate that the QAPAF is a psychometrically sound tool for assessing perceptions at the population level [27]. Temporal stability was assessed in a subsample of 25 participants who completed the questionnaire again after a two-week interval. Test and retest totals were identical for all participants (Wilcoxon signed-rank test: Z = 0, *p* = 1.000; effect size r = 0.00), indicating point-by-point agreement over the two-week interval. Because there were no between-time differences, uncertainty for ρ and ICC could not be estimated, which limits assessment of measurement error. Larger samples and settings with natural within-person fluctuation would yield more informative reliability estimates. We also recommend complementing rank-based tests with absolute-agreement metrics and Bland–Altman analysis in future work. These results may also reflect the inherently dynamic nature of food perception, which can be influenced by exposure to media, marketing strategies, public health campaigns, or personal experiences [17,45,46]. Still, the observed stability supports the potential utility of the QAPAF for monitoring perception changes over time, particularly in the context of educational or behavioural interventions [47].

There is no single gold-standard instrument for functional food perception against which criterion validity could be established. We therefore followed recognised standards for health status questionnaires, assessing content validity, internal consistency, structural validity by exploratory factor analysis, and test–retest reliability in line with published quality criteria (e.g., Terwee and colleagues [40]). The QAPAF is feasible for population studies because it comprises 17 items on a single page, uses a five-point response scale with a neutral option, allows simple total scoring from 17 to 85, is generic rather than disease-specific, and was written for lay respondents [26,27]. These features support its use in surveillance and evaluation settings and facilitate administration alongside sociodemographic and lifestyle measures.

### 4.2. Application of the QAPAF in the Portuguese Population

Study 2 applied the QAPAF to a broader sample to explore perceptions of functional foods and their association with sociodemographic and lifestyle variables. The results revealed a generally favourable perception of functional foods. Most participants agreed that such products should be consumed as part of a balanced diet and rejected the idea that they are only useful for specific groups such as older adults or individuals with chronic conditions [48]. However, the data also revealed persistent uncertainty, particularly in relation to safety, efficacy, and marketing claims. Neutral responses to statements about the ability of functional foods to reverse dietary damage or the reliability of advertised benefits suggest a degree of public scepticism. These perceptions mirror those found in other European studies, where concerns about product processing, artificiality, and the credibility of health claims are common [7,12,16,25]. Such scepticism may act as a barrier to the adoption of functional foods and should be addressed through transparent labelling and clearer communication [49]. Participants with correct knowledge about what constitutes a functional food were more likely to express trust in professional recommendations and to reject misconceptions. This highlights the importance of food literacy, not only as a knowledge domain but also as a determinant of confidence, trust, and intention to consume [22,24]. Educational interventions that improve understanding of functional food definitions and health benefits may therefore contribute to more informed dietary choices [13,32]. The study also found that individuals with higher levels of education, as well as those who abstain from smoking or alcohol, had significantly higher QAPAF scores. These associations suggest that the acceptance of functional foods is related to broader health-oriented behaviours and to individual motivation for health maintenance [19,28]. No differences were found according to sex, income, or BMI, indicating that attitudes may depend more on behavioural and cognitive factors than on demographic traits [20]. These findings have important implications for public health and nutrition policy. As functional foods continue to expand in the European market, there is a growing need to ensure that consumers can critically interpret health claims, understand product functionality, and make choices that align with evidence-based dietary guidelines [15,38,50]. Drawing on our prior findings (Oliveira et al. [51]), the QAPAF may help identify subgroups with low confidence or limited understanding and guide tailored health communication and educational programmes, pending confirmation in more diverse samples.

In addition, the tool could support the integration of functional foods into culturally appropriate dietary models, such as the Mediterranean diet. By identifying barriers and facilitators to acceptance, researchers and policymakers can better design interventions that promote both individual and public health outcomes [14,29,52]. Further validation of the QAPAF in other cultural and linguistic contexts is recommended. Longitudinal studies and applications in intervention settings will help establish the tool’s predictive validity and responsiveness to change. Exploring its association with actual dietary intake and health outcomes would also expand its utility for guiding population-based nutrition strategies [53].

### 4.3. Implications for Nutrition and Public Health

The QAPAF provides a practical and validated tool for assessing public perceptions of functional foods in adult populations. Its application in this study revealed that conceptual understanding and health-oriented behaviours are key predictors of positive attitudes toward functional foods [2,8,18]. These insights may support the development of more effective nutrition education strategies, especially in populations with lower health literacy [21,54]. By identifying groups with limited trust or misperceptions, public health professionals can design targeted interventions that clarify the role of functional foods within a healthy diet [11,55]. The instrument may also be useful in clinical nutrition settings to explore patient attitudes and inform personalised counselling [56]. From a policy perspective, the findings highlight the importance of transparent communication and regulation of health claims on food products. Ensuring that functional foods are not misunderstood or overestimated in their benefits is essential for promoting realistic and evidence-based dietary choices [31]. Furthermore, the QAPAF can be integrated into monitoring and evaluation frameworks to track changes in public perception over time, particularly in response to national campaigns, product reformulations, or regulatory changes. Its utility in future intervention research may also help assess the impact of food literacy programmes on consumer attitudes and behaviours [57].

### 4.4. Strengths and Limitations

One of the main strengths of this study lies in the robust psychometric re-evaluation of a culturally adapted instrument designed to assess consumer perceptions of functional foods. The QAPAF was tested in a real-world setting with a relatively large and diverse adult sample, enhancing its applicability in population-level research and nutritional surveillance. The multidimensional structure of the instrument allows for a more comprehensive understanding of attitudes and beliefs, extending beyond knowledge to include trust, perceived necessity, and safety concerns [27]. Another strength is the inclusion of relevant behavioural and lifestyle variables, such as smoking status, alcohol consumption, and adherence to healthier dietary behaviours. These factors are often underrepresented in studies of consumer perception but proved to be significantly associated with QAPAF scores, offering insight into how functional food acceptance fits within broader health-related patterns [30].

However, several limitations should be acknowledged. Generalisability is limited by the convenience sampling and by the predominance of female and highly educated participants; external validation in more diverse and preferably probability-based samples is warranted. The sample was recruited through convenience methods and was not representative of the general Portuguese population. Participants were predominantly female and highly educated, which may have influenced the overall attitudes and reduced the variability in perceptions [58]. Additionally, data collection relied on self-reported measures, including height, weight, and health status. This may introduce reporting bias or inaccuracies in BMI estimation [41]. The online format of the survey may have excluded individuals with limited digital access or literacy, potentially affecting sample diversity. Lastly, test–retest reliability was assessed in a relatively small subsample (*n* = 25), which constrains the precision and generalisability of the stability estimates. In this subsample, agreement across time points was perfect (Spearman ρ = 1.00; two-way mixed ICC, absolute agreement, single measures = 1.00), and all paired differences were zero (Wilcoxon Z = 0, *p* = 1.000; Bland–Altman bias = 0.00 with 95% limits of agreement 0.00 to 0.00). While this supports temporal stability over the two-week interval, the absence of between-time variation precluded estimating uncertainty for ρ and ICC. Confirmation in larger and more diverse retest cohorts is warranted [59]. Nonetheless, the chosen sample size is consistent with that used in preliminary psychometric studies of similar instruments [40,60]. Future work should also examine measurement invariance and predictive validity across key sociodemographic strata to strengthen external validity.

## 5. Conclusions

The QAPAF proved to be a valid and reliable instrument for assessing perceptions of functional foods in the Portuguese adult population. Its four-factor structure reflects key dimensions of consumer beliefs and attitudes, including confidence in health professionals, safety concerns, misconceptions, and general trust. The application of the QAPAF revealed significant associations with education level and health-related behaviours, suggesting its potential utility in identifying target groups for nutrition education. As functional foods continue to gain prominence in public health strategies, the QAPAF may serve as a valuable tool to inform and tailor food literacy interventions, supporting more informed and health-conscious food choices. Further research is warranted to confirm its applicability across different cultural and demographic contexts.

## Figures and Tables

**Table 1:** Total variance explained by the four retained factors of the Questionnaire for the Assessment of Perception of Functional Foods (*n* = 343).

Component	Initial Eigenvalues			Extraction Sums of Squared Loadings			Rotation Sums of Squared Loadings
	Total	% of Variance	Cumulative %	Total	% of Variance	Cumulative %	Total
1	4.547	26.746	26.746	4.547	26.746	26.746	3.996
2	2.578	15.167	41.912	2.578	15.167	41.912	3.101
3	1.838	10.813	52.725	1.838	10.813	52.725	2.213
4	1.029	6.051	58.776	1.029	6.051	58.776	1.318
5	0.919	5.405	64.182				
6	0.826	4.859	69.040				
7	0.761	4.475	73.516				
8	0.715	4.204	77.720				
9	0.622	3.659	81.378				
10	0.595	3.502	84.880				
11	0.477	2.806	87.686				
12	0.460	2.703	90.390				
13	0.403	2.369	92.759				
14	0.371	2.180	94.939				
15	0.332	1.953	96.893				
16	0.283	1.664	98.557				
17	0.245	1.443	100.000				

Data are presented as eigenvalues, explained variance (%), and cumulative variance (%) for each extracted component. Components were retained based on eigenvalues greater than 1. No rotation method was applied. The four retained components accounted for 58.8% of the total explained variance.

**Table 2:** Test–retest agreement of Questionnaire for the Assessment of Perception of Functional Foods total score (*n* = 25).

Item	Test Median (IQR)	Retest Median (IQR)	Z	*p*-Value
QAPAF total	61.0 (55.0; 63.0)	61.0 (55.0; 63.0)	0.000	1.000

Data are presented as medians with interquartile ranges [median (IQR)]. Comparisons between time points were performed using the Wilcoxon Signed-Rank Test. Statistical significance was defined as *p* < 0.05.

**Table 3:** Sociodemographic and Lifestyle Characteristics of the Participants According to Biological Sex and Understanding of Functional Foods (*n* = 343).

Sociodemographic and Lifestyle Characteristics	Total	Sex		*p*	What Do You Understand by Functional Foods or What Do You Think Functional Foods Can Be?		*p*
		Male	Female		Correct	Incorrect	
Sex, % (*n*)							
Female	73.8 (253)	---	---	---	40.7% (103)	59.3% (150)	0.244
Male	26.2 (90)	---	---	---	47.8% (43)	52.2% (47)	
Age (years), median (IQR)	31.0 (23.0; 45.0)	37.0 (23.0; 47.3)	29.0 (22.0; 44.0)	0.041 *	29.0 (22.8; 47.0)	32.0 (22.5; 25.6)	0.891
Marital status, % (*n*)							
Married	62.4 (214)	52.2 (47)	66.0 (167)	0.020 *	45.8% (98)	54.2% (116)	0.119
Single/Divorced/Widowed	37.6 (129)	47.8 (43)	34.0 (86)		37.2% (48)	62.8% (81)	
Region of residence, % (*n*)							
Lives in Lisbon	42.9 (147)	53.3 (48)	39.1 (99)	0.019 *	39.5% (58)	60.5% (89)	0.313
Lives in other regions	57.1 (196)	46.7 (42)	60.9 (154)		44.9% (88)	55.1% (108)	
Type of residential area, % (*n*)							
Rural area	17.5 (60)	13.3 (12)	19.0 (48)	0.227	41.7% (25)	58.3% (35)	0.877
Urban area	82.5 (283)	86.7 (78)	81.0 (205)		42.8% (121)	57.2% (162)	
Educational level, % (*n*)							
Up to 12th grade	38.5 (132)	32.2 (29)	40.7 (103)	0.155	47.7% (63)	52.3% (69)	0.126
Higher education	61.5 (211)	67.8 (61)	59.3 (150)		39.3% (83)	60.7% (128)	
Field of study or professional area, % (*n*) (*n* = 213)							
Health-related professional area	26.5 (91)	29.5 (18)	48.0 (73)	0.014 *	37.4% (34)	62.6% (57)	0.172
Other professional area	35.6 (122)	70.5 (43)	52.0 (79)		46.7% (57)	53.3% (65)	
Body Mass Index Classification, % (*n*) (*n* = 275)							
Low and Normal weight	54.2 (186)	58.6 (41)	70.7 (145)	0.060	40.9% (76)	59.1% (110)	0.642
Overweight	25.9 (89)	41.4 (29)	29.3 (60)		43.8% (39)	56.2% (50)	
Body Mass Index (kg/m^2^), median (IQR) (*n* = 275)	22.9 (20.7; 25.6)	24.6 (21.8; 26.4)	22.5 (20.6; 25.2)	0.004 *	22.8 (20.8;25.3)	23.0 (20.7; 25.9)	0.881
Chronic condition or disease, % (*n*)							
No chronic condition	48.4 (166)	70.0 (49)	57.1 (117)	0.056	39.2% (65)	60.8% (101)	0.269
With chronic condition	31.8 (109)	30.0 (21)	42.9 (88)		45.9% (50)	54.1% (59)	
Smoking habits, % (*n*) (*n* = 248)							
Smoker	10.8 (37)	21.1 (12)	13.1 (25)	0.139	48.6% (18)	51.4% (19)	0.431
Non-smoker	61.5 (211)	78.9 (45)	86.9 (166)		41.7% (88)	58.3% (123)	
Alcohol consumption, % (*n*) (*n* = 248)							
Consumes alcoholic beverages	35.0 (120)	61.4 (35)	44.5 (85)	0.025 *	45.0% (54)	55.0% (66)	0.486
Does not consume alcoholic beverages	37.3 (128)	38.6 (22)	55.5 (106)		40.6% (52)	59.4% (76)	
Caffeine consumption, % (*n*) (*n* = 248)							
Consumes coffee or energy drinks	56.9 (195)	84.2 (48)	77.0 (147)	0.241	41.5% (81)	58.5% (114)	0.462
Does not consume coffee or energy drinks	15.5. (53)	15.8 (9)	23.0 (44)		47.2% (25)	52.8% (28)	
Monthly Family Income (€), median (IQR) (*n* = 203)	2400.0 (1500.0; 3000.0)	2500.0 (1800.0; 3575.0)	2000.0 (1500.0; 3000.0)	0.030 *	2400.0 (1500.0; 3000.0)	2350.0 (1500.0; 3000.0)	0.594

Data are presented as percentages and counts [% (*n*)] for categorical variables, and as medians with interquartile ranges [median (IQR)] for continuous variables. Comparisons between groups according to biological sex and understanding of functional foods were performed using the Pearson’s Chi-Square Test or Fisher’s Exact Test for categorical variables, and the Mann–Whitney U Test for continuous variables. * Statistical significance was defined as *p* < 0.05. Correct understanding: Foods that promote health and well-being and/or reduce the risk of certain diseases. Incorrect understanding: Foods in the form of tablets, capsules, or other dietary supplements; or foods perceived merely as healthy items that help maintain normal body functions.

**Table 4:** Distribution of Participants’ Responses to Statements about Functional Foods (*n* = 343).

Statements		Strongly Disagree	Disagree	Neither Agree nor Disagree	Agree	Strongly Agree	*p*
Functional foods do not replace a healthy diet but should be consumed as part of a varied diet.	Total	2.0% (7)	6.4% (22)	25.7% (88)	39.7% (136)	26.2% (90)	---
Functional foods are useless for a healthy person.	Total	30.9% (106)	38.8% (133)	22.7% (78)	5.2% (18)	2.3% (8)	---
Functional foods can repair damage caused by an unhealthy diet.	Total	9.3% (32)	16.3% (56)	39.7% (136)	31.8% (109)	2.9% (10)	---
Functional foods do not taste good.	Total	22.4% (77)	35.0% (120)	34.4% (118)	6.4% (22)	1.7% (6)	---
Functional foods are unnecessary.	Correct	56.8% (88)	15.5% (24)	7.1% (11)	5.8% (9)	14.8% (23)	0.027 *
	Incorrect	0.5% (1)	8.8% (19)	12.5% (27)	50.9% (110)	27.3% (59)	
	Total	32.1% (110)	39.4% (135)	24.2% (83)	3.8% (13)	0.6% (2)	---
Advertisements that claim benefits of functional foods are false.	Total	14.3% (49)	35.6% (122)	41.1% (141)	8.2% (28)	0.9% (3)	---
Functional foods are only for the elderly, the sick, or children.	Correct	54.9% (128)	25.3% (59)	0.9% (2)	1.7% (4)	17.2% (40)	0.005 *
	Incorrect	12.6% (47)	3.8% (14)	50.7% (189)	10.5% (39)	22.5% (84)	
	Total	37.0% (127)	38.5% (132)	19.8% (68)	4.1% (14)	0.6% (2)	---
Functional foods may have undesirable effects.	Total	8.2% (28)	23.0% (79)	44.0% (151)	20.7% (71)	4.1% (14)	---
Functional foods are capable of improving my well-being.	Total	2.6% (9)	6.1% (21)	32.9% (113)	46.9% (161)	11.4% (39)	---
It is safe to use functional foods.	Total	1.7% (6)	3.5% (12)	28.3% (97)	52.2% (179)	14.3% (49)	---
Functional foods are a passing trend.	Total	18.7% (64)	37.6% (129)	36.2% (124)	6.1% (21)	1.5% (5)	---
The safety of functional foods is well studied.	Total	1.5% (5)	9.0% (31)	47.5% (163)	35.0% (120)	7.0% (24)	---
Excessive consumption of functional foods is harmful.	Total	6.1% (21)	17.2% (59)	40.5% (139)	27.1% (93)	9.0% (31)	---
Functional foods are more expensive.	Total	6.7% (23)	19.0% (65)	42.0% (144)	26.5% (91)	5.8% (20)	---
Only foods that have health benefit claims on the label are considered functional.	Total	16.6% (57)	33.2% (114)	37.9% (130)	9.0% (31)	3.2% (11)	---
I believe in the effect of functional foods if a health professional (doctor, nutritionist, etc.) recommends the product.	Correct	19.7% (51)	5.4% (14)	32.4% (84)	10.8% (28)	31.7% (82)	0.019 *
	Incorrect	2.0% (5)	53.1% (136)	23.8% (61)	1.6% (4)	19.5% (50)	
	Total	2.3% (8)	8.2% (28)	29.7% (102)	45.8% (157)	14.0% (48)	---
Functional foods truly have the health benefits they claim.	Total	3.8% (13)	7.0% (24)	48.4% (166)	35.3% (121)	5.5% (19)	---

This table shows the percentage and absolute distribution of participants’ responses to several statements concerning functional foods, based on a 5-point Likert scale: Strongly Disagree, Disagree, Neither Agree nor Disagree, Agree, Strongly Agree. For some statements, results are disaggregated by knowledge level (correct or incorrect response). Values are presented as percentages followed by the number of responses in parentheses. The “*p*” column indicates the statistical significance of the association between knowledge level and response distribution (Chi-square test). * Statistical significance was defined as *p* < 0.05.

**Table 5:** Association between Sociodemographic and Lifestyle Characteristics and Questionnaire for the Assessment of Perception of Functional Foods Score.

Sociodemographic and Lifestyle Characteristics	*n*	QAPAF	*p*
		Median (IQR)	
Sex			
Female	253	59.0 (54.5; 63.0)	0.711
Male	90	58.0 (55.0; 63.3)	
Marital status			
Married	129	59.0 (54.5; 62.5)	0.903
Single/Divorced/Widowed	214	59.0 (55.0; 64.0)	
Region of residence			
Lives in Lisbon	147	59.0 (53.0; 63.0)	0.634
Lives in other regions	196	59.0 (55.0; 62.8)	
Type of residential area			
Rural area	60	59.0 (55.0; 62.0)	0.907
Urban area	283	59.0 (54.0; 64.0)	
Educational level			
Up to 12th grade	132	58.0 (52.0; 61.8)	0.010 *
Higher education	211	59.0 (55.0; 64.0)	
Field of study or professional area (*n* = 213)			
Health-related professional area	91	60.0 (56.0; 64.0)	0.329
Other professional area	122	59.0 (55.0; 62.3)	
Body Mass Index Classification (*n* = 275)			
Low and Normal weight	186	59.0 (53.0; 62.0)	0.409
Overweight	89	59.0 (54.5; 64.0)	
Chronic condition or disease (*n* = 275)			
No chronic condition	166	59.0 (54.0; 63.0)	0.329
With chronic condition	109	58.0 (53.0; 63.0)	
Smoking habits (*n* = 248)			
Smoker	37	57.0 (50.5; 60.0)	0.008 *
Non-smoker	211	59.0 (54.0; 64.0)	
Alcohol consumption (*n* = 248)			
Consumes alcoholic beverages	120	57.0 (53.0; 61.0)	0.020 *
Does not consume alcoholic beverages	128	59.0 (53.3; 64.0)	
Caffeine consumption (*n* = 248)			
Consumes coffee or energy drinks	195	59.0 (53.0; 62.0)	0.230
Does not consume coffee or energy drinks	53	59.0 (54.0; 65.0)	
Age, median (years)	343		(r: −0.008; *p*: 0.883)
Body Mass Index (kg/m^2^)	275		(r: 0.050; *p*: 0.407)
Monthly Family Income (€)	203		(r: −0.058; *p*: 0.408)

Data are presented as medians with interquartile ranges [median (IQR)] for continuous variables. Comparisons between groups were performed using the Mann–Whitney U Test for categorical variables with two levels. For continuous variables such as age, body mass index (BMI), and monthly family income, Spearman’s correlation coefficients (r) and corresponding *p*-values are reported. * Statistical significance was defined as *p* < 0.05.

## Data Availability

The original contributions presented in this study are included in the article/Supplementary Material. Further inquiries can be directed to the corresponding authors.

## Supplementary Materials

The following supporting information can be downloaded at: https://www.mdpi.com/article/10.3390/nu17182938/s1, Table S1. Items of the Questionnaire for the Assessment of Perception of Functional Foods and Likert-scale response options; Table S2. Communalities of the Questionnaire for the Assessment of Perception of Functional Foods items based on principal component analysis (n = 343); Table S3. Item-total statistics and Cronbach’s alpha if item deleted for the Questionnaire for the Assessment of Perception of Functional Foods (n = 343); Table S4: Oblique four-component solution for the QAPAF (n = 343, listwise complete): pattern matrix, structure matrix, and factor correlation matrix. Extraction by principal components. Rotation by direct oblimin (delta = 0) with Kaiser normalization; Figure S1. The scree plot displays the eigenvalues associated with each principal component. The elbow observed after the fourth component supports the retention of four factors; Figure S2: Scree plot for the QAPAF item set (n = 343). Extraction: principal components. The elbow at the fourth component, together with eigenvalues greater than 1, supports retaining four components; Figure S3: Bland–Altman plot for the QAPAF total score (*n* = 25). The mean difference (bias) was 0.00 and the 95% limits of agreement were 0.00 to 0.00, because all paired differences were zero. Points lie on the horizontal line at 0, indicating perfect point-by-point agreement between test and retest.
