# Supplementary material for: Validation and Application of an Online Self-Assessment Questionnaire for the Assessment of Perception of Functional Foods: A Cross-Sectional Psychometric Study in Adults
Source: Nutrients. 2025 Sep 12;17(18):2938. doi: 10.3390/nu17182938 (PMC12472828; doi:10.3390/nu17182938)
Supplement: Supplementary file 1 [file nutrients-17-02938-s001.zip › nutrients-3849743-supplementary.pdf]

Table S1. Items of the Questionnaire for the Assessment of Perception of Functional Foods and Likert-scale response options.

Nº	Item (Português)	Item (English)	Discordo Muito / Strongly Disagree	Discordo / Disagree	Nem Concordo nem Discordo / Neither Agree nor Disagree	Concordo / Agree	Concordo Muito / Strongly Agree
1	Os alimentos funcionais não substituem uma alimentação saudável, mas devem ser consumidos como parte de uma alimentação variada.	Functional foods do not replace a healthy diet but should be consumed as part of a varied diet.					
2	Os alimentos funcionais são inúteis para uma pessoa saudável.	Functional foods are useless for a healthy person.					
3	Os alimentos funcionais podem reparar os danos causados por uma alimentação pouco saudável.	Functional foods can repair damage caused by an unhealthy diet.					
4	Os alimentos funcionais não têm um sabor agradável.	Functional foods do not taste good.					
5	Os alimentos funcionais são desnecessários.	Functional foods are unnecessary.					
6	Os anúncios que referem benefícios dos alimentos funcionais são falsos.	Advertisements that claim benefits of functional foods are false.					
7	Os alimentos funcionais são apenas para idosos, doentes ou crianças.	Functional foods are only for the elderly, the sick, or children.					
8	Os alimentos funcionais podem ter efeitos indesejáveis.	Functional foods may have undesirable effects.					

9	Os alimentos funcionais são capazes de melhorar o meu bem-estar.	Functional foods are capable of improving my well-being.					
10	É seguro utilizar alimentos funcionais.	It is safe to use functional foods.					
11	Os alimentos funcionais são uma moda que vai passar.	Functional foods are a passing trend.					
12	A segurança dos alimentos funcionais está bem estudada.	The safety of functional foods is well studied.					
13	Os alimentos funcionais em excesso são prejudiciais.	Excessive consumption of functional foods is harmful.					
14	Os alimentos funcionais são mais caros.	Functional foods are more expensive.					
15	Os únicos alimentos funcionais são aqueles cujos rótulos alegam benefícios de saúde.	Only foods that have health benefit claims on the label are considered functional.					
16	Acredito no efeito dos alimentos funcionais se um técnico de saúde (médico, nutricionista, etc.) me recomendar o produto.	I believe in the effect of functional foods if a health professional (doctor, nutritionist, etc.) recommends the product.					
17	Os alimentos funcionais têm, de facto, os benefícios para a saúde que são anunciados.	Functional foods truly have the health benefits they claim.					

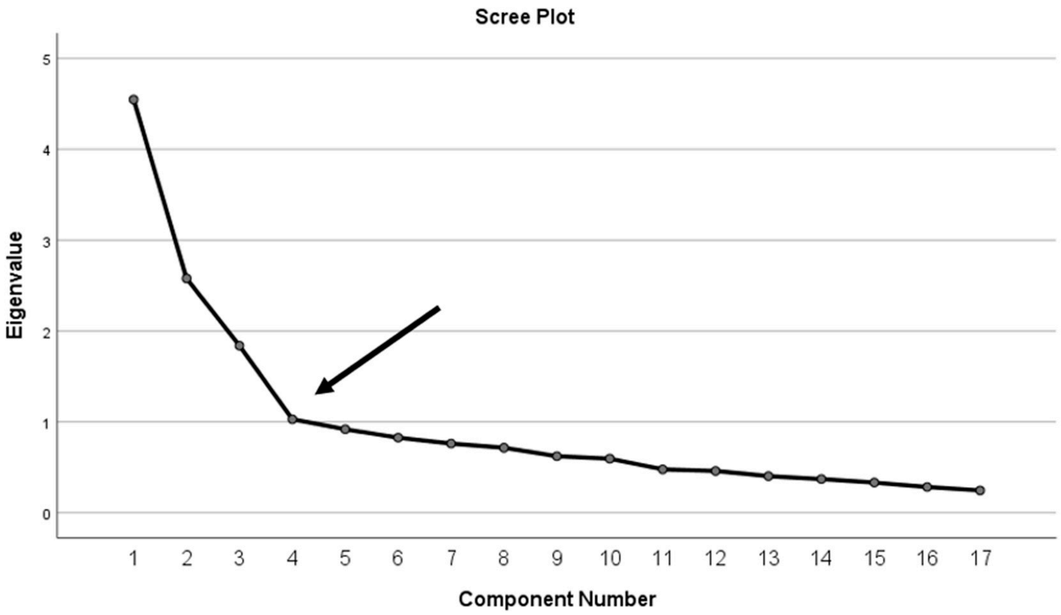

Figure S1. The scree plot displays the eigenvalues associated with each principal component. The elbow observed after the fourth component supports the retention of four factors.

Table S2. Communalities of the Questionnaire for the Assessment of Perception of Functional Foods items based on principal component analysis (n = 343).

Item	Statement	Initial	Extraction
1	Functional foods do not replace a healthy diet but should be consumed as part of a varied diet.	1.000	0.616
2	Functional foods are useless for a healthy person.	1.000	0.639
3	Functional foods can repair damage caused by an unhealthy diet.	1.000	0.745
4	Functional foods do not taste good.	1.000	0.513
5	Functional foods are unnecessary.	1.000	0.733
6	Advertisements that claim benefits of functional foods are false.	1.000	0.488
7	Functional foods are only for the elderly, the sick, or children.	1.000	0.675
8	Functional foods may have undesirable effects.	1.000	0.673
9	Functional foods are capable of improving my well-being.	1.000	0.660
10	It is safe to use functional foods.	1.000	0.722
11	Functional foods are a passing trend.	1.000	0.474
12	The safety of functional foods is well studied.	1.000	0.679
13	Excessive consumption of functional foods is harmful.	1.000	0.659
14	Functional foods are more expensive.	1.000	0.446
15	Only foods that have health benefit claims on the label are considered functional.	1.000	0.506
16	I believe in the effect of functional foods if a health professional (doctor, nutritionist, etc.) recommends the product.	1.000	0.464
17	Functional foods truly have the health benefits they claim.	1.000	0.301

Legend: Extraction communalities represent the proportion of each item's variance explained by the retained components. Values ≥ 0.40 are considered acceptable.

Table S3. Item-total statistics and Cronbach's alpha if item deleted for the Questionnaire for the Assessment of Perception of Functional Foods (n = 343).

Item	Scale Mean if Item Deleted	Scale Variance if Item Deleted	Corrected Item-Total Correlation	Cronbach's Alpha if Item Deleted
1	55.01	37.941	0.273	0.691
2	54.92	33.879	0.641	0.647
3	55.80	40.941	0.015	0.720
4	55.12	36.546	0.408	0.676
5	54.84	34.020	0.715	0.643
6	55.28	35.699	0.546	0.662
7	54.75	35.487	0.554	0.661
8	55.72	38.098	0.261	0.692
9	55.24	37.288	0.384	0.679
10	55.08	36.639	0.491	0.670
11	55.16	36.067	0.484	0.668
12	55.45	38.202	0.329	0.686
13	55.66	44.381	-0.245	0.748
14	55.88	39.152	0.163	0.703
15	55.31	37.467	0.307	0.687
16	56.43	45.147	-0.317	0.748
17	55.50	38.923	0.239	0.694

Legend: This table presents the corrected item-total correlations, the scale mean and variance if each item were deleted, and the corresponding Cronbach's alpha value. These indicators allow for evaluation of each item's contribution to the internal consistency of the scale. Items 13 and 16 exhibited negative item-total correlations, suggesting weak alignment with the overall construct. However, they were retained to preserve the content validity of the original instrument.

Table S4. Oblique four-component solution for the QAPAF (n = 343, listwise complete): pattern matrix, structure matrix, and factor correlation matrix. Extraction by principal components. Rotation by direct oblimin (delta = 0) with Kaiser normalization.

Item	h^2	C 1	C 2	C 3	C 4
1	0.616	0.321	-0.310	0.642	0.040
2	0.639	0.723	-0.185	-0.106	0.049
3	0.745	-0.159	-0.164	-0.106	0.823
4	0.513	0.616	0.015	-0.084	-0.352
5	0.733	0.810	-0.153	0.014	0.078
6	0.488	0.588	-0.213	-0.177	-0.034
7	0.675	0.788	-0.065	0.233	-0.195
8	0.673	0.227	-0.150	-0.741	-0.064
9	0.660	0.159	-0.571	0.119	0.443
10	0.722	0.194	-0.776	-0.010	0.101
11	0.474	0.611	-0.058	-0.226	-0.026
12	0.679	0.000	-0.804	-0.225	-0.155
13	0.659	0.010	0.132	0.806	-0.064
14	0.446	0.330	0.295	-0.471	0.149
15	0.506	0.642	0.359	0.120	0.253
16	0.464	-0.022	0.647	-0.165	-0.021
17	0.301	0.019	-0.497	0.000	0.173

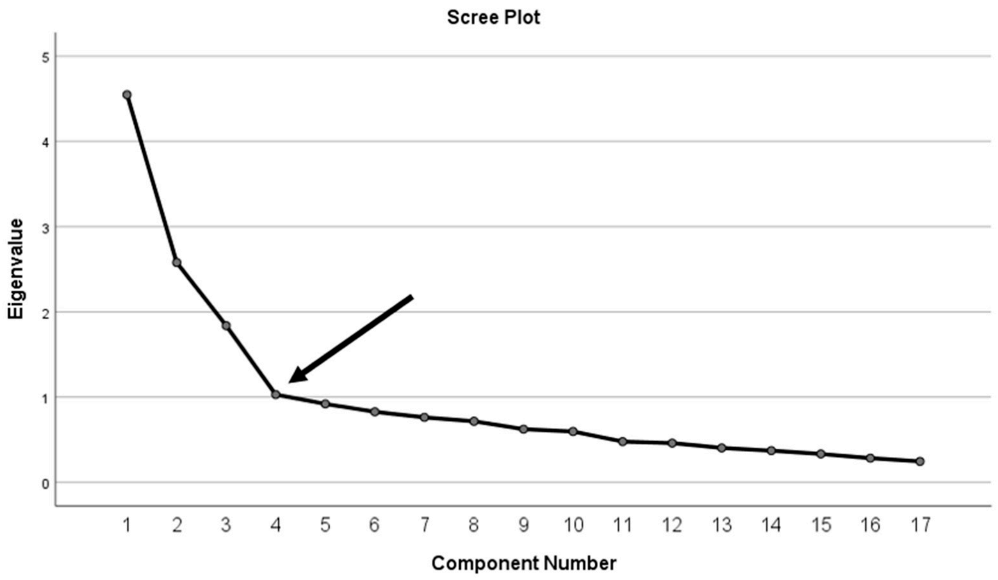

Figure S2. Scree plot for the QAPAF item set (n = 343). Extraction: principal components. The elbow at the fourth component, together with eigenvalues greater than 1, supports retaining four components.

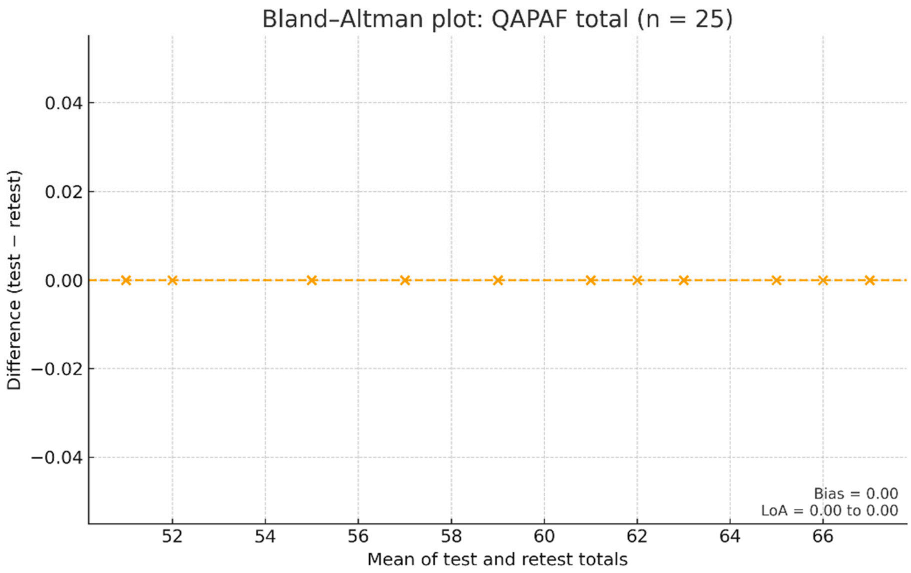

Figure S3. Bland–Altman plot for the QAPAF total score (n = 25). The mean difference (bias) was 0.00 and the 95% limits of agreement were 0.00 to 0.00, because all paired differences were zero. Points lie on the horizontal line at 0, indicating perfect point-by-point agreement between test and retest.
